# Targeting metabolic vulnerabilities to overcome resistance to therapy in acute myeloid leukemia

**DOI:** 10.20517/cdr.2023.12

**Published:** 2023-08-17

**Authors:** Priyanka Sharma, Gautam Borthakur

**Affiliations:** Department of Leukemia, Section of Molecular Hematology and Therapeutics, The University of Texas MD Anderson Cancer Center, Houston, TX 77030, USA.

**Keywords:** OXPHOS, DHODH, leukemia stem cells, mesenchymal stromal cells, IDH

## Abstract

Malignant hematopoietic cells gain metabolic plasticity, reorganize anabolic mechanisms to improve anabolic output and prevent oxidative damage, and bypass cell cycle checkpoints, eventually outcompeting normal hematopoietic cells. Current therapeutic strategies of acute myeloid leukemia (AML) are based on prognostic stratification that includes mutation profile as the closest surrogate to disease biology. Clinical efficacy of targeted therapies, e.g., agents targeting mutant FMS-like tyrosine kinase 3 (FLT3) and isocitrate dehydrogenase 1 or 2, are mostly limited to the presence of relevant mutations. Recent studies have not only demonstrated that specific mutations in AML create metabolic vulnerabilities but also highlighted the efficacy of targeting metabolic vulnerabilities in combination with inhibitors of these mutations. Therefore, delineating the functional relationships between genetic stratification, metabolic dependencies, and response to specific inhibitors of these vulnerabilities is crucial for identifying more effective therapeutic regimens, understanding resistance mechanisms, and identifying early response markers, ultimately improving the likelihood of cure. In addition, metabolic changes occurring in the tumor microenvironment have also been reported as therapeutic targets. The metabolic profiles of leukemia stem cells (LSCs) differ, and relapsed/refractory LSCs switch to alternative metabolic pathways, fueling oxidative phosphorylation (OXPHOS), rendering them therapeutically resistant. In this review, we discuss the role of cancer metabolic pathways that contribute to the metabolic plasticity of AML and confer resistance to standard therapy; we also highlight the latest promising developments in the field in translating these important findings to the clinic and discuss the tumor microenvironment that supports metabolic plasticity and interplay with AML cells.

## INTRODUCTION

Acute myeloid leukemia (AML) is a cancer derived from the myeloid lineage of blood cells. It is characterized by overproduction of leukemic blasts and maturation arrest. With approximately 120,000 cases a year globally, AML is the most common type of acute leukemia in adults^[1]^. Although conventional chemotherapy is known to eliminate bulk tumor cells, its utility is limited in AML, as older patients lack tolerance to chemotherapy and the drug may lack efficacy at eliminating leukemia stem cells (LSCs). Residual clones that survive chemotherapy lead to disease relapse^[2]^. Moreover, AML exhibits extensive genetic heterogeneity, as it often harbors a complex mixture of heterogenous subclones that possess genetic aberrations in diverse driver genes, with distinct clonal evolutionary patterns existing in single individuals. Cytogenetic abnormalities and acquired somatic mutations are used as surrogate markers for risk assessment and some mutations can be specifically targeted. However, the utility of therapies that target mutations viz. FMS-like tyrosine kinase 3 (FLT3) and isocitrate dehydrogenase enzyme 2 (IDH2) are limited to the context of the particular mutation.

Cancer cells are known to reprogram their metabolism to support their survival and proliferation; this was recently recognized as a hallmark of cancer^[3]^. AML cells exhibit unique metabolic dependencies compared with those of their normal counterparts, the noncancerous blasts. Hematopoietic stem cells (HSCs) use mitochondrial oxidative phosphorylation (OXPHOS) during differentiation to meet their higher energy requirements; however, they use anaerobic glycolysis for their routine energy needs and generally avoid aerobic mitochondrial OXPHOS^[4]^. Of note, mitochondrial OXPHOS generates reactive oxygen species (ROS), which are deleterious for HSCs. In fact, ROS functions as a signaling mediator in the crosstalk between metabolism and stem cell fate decisions by inhibiting the repopulating capacity of HSCs^[5]^. ROS-high-HSCs are characterized by low self-renewal capacity and high myeloid differentiation capacity compared to ROS-low HSCs.

Upon cell division, HSCs have fate choices when they undergo cell division; they either undergo self-renewal, wherein they produce new HSCs, or differentiate, wherein they produce cells that mature into committed cells. On the basis of the status of the daughter cells, HSC division can occur in one of the following ways: (1) asymmetric division, which maintains the HSC pool, in which one daughter cell remains as stem cell while the other differentiates; (2) symmetric commitment, in which both daughter cells differentiate (stem cell exhaustion); or (3) symmetric division, in which two daughter stem cells are produced, which helps in HSC expansion*.* Interestingly, mitochondria distribution during stem cell division appears to be crucial to determining the fate of HSCs. Of note, mitochondrial division is asymmetrical during stem cell division and daughter cells that receive fewer old mitochondria maintain stem cell traits^[6]^. Notably, through a ROS-mediated physiological process, changes in mitochondrial dynamics regulate stem cell fate decisions, triggering a dual program to suppress self-renewal and promote differentiation^[6,7]^. Evasion from mitochondrial OXPHOS and reliance on glycolysis help prevent HSC pool exhaustion. In contrast, LSCs rely on mitochondrial OXPHOS to generate high-energy compounds while maintaining ROS levels at non-toxic levels by employing a wide repertoire mechanism for ROS mitigation^[8]^. Therefore, it may be possible to target leukemic cells differently based on their metabolic needs for therapeutic purposes.

**Figure 1:**
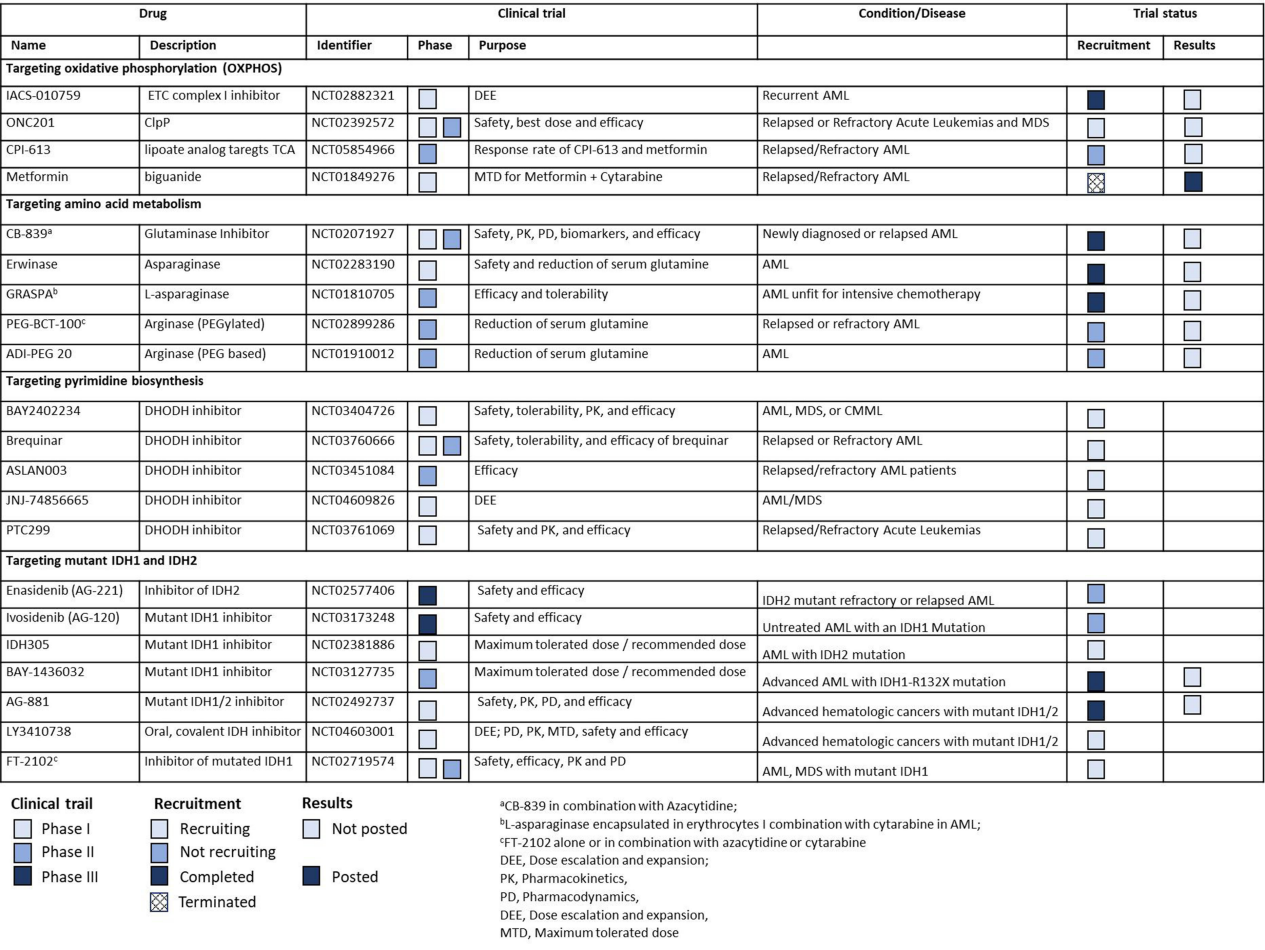
List of clinical trials for metabolic inhibitors in AML. AML: Acute myeloid leukemia; ClpP: caseinolytic protease proteolytic subunit; CMML: chronic myelomonocytic leukemia; DHODH: dihydroorotate dehydrogenase; ETC: electron transport chain; IDH: isocitrate dehydrogenase enzyme; MDS: myelodysplastic syndromes; OXPHOS: oxidative phosphorylation; TCA: tricarboxylic acid cycle.

In this review, we discuss the role of cancer metabolic pathways in AML development that contribute to the metabolic plasticity of the disease and confer resistance to standard therapy. We also highlight the latest developments in the field including clinical trials for translating metabolic inhibitors to clinic [Figure 1] and discuss the role of tumor microenvironment and extracellular vesicles in supporting metabolic plasticity and their interplay with AML cells.

## TARGETING MITOCHONDRIAL OXPHOS

AML cells are more sensitive to mitochondria-targeted drugs, likely because despite having increased respiratory activity, their mitochondria have lower coupling efficiency and spare reserve capacity due to proton leak^[9]^. In different cancers, including AML, depletion of mtDNA results in OXPHOS deficiency. OXPHOS-deficient cancer cells fail to form tumors unless mtDNA is restored from stromal cells via the horizontal transfer of whole mitochondria^[10-13]^, suggesting that functional OXPHOS is essential for cancer development, although it is not clear which component of OXPHOS contributes the most to tumor formation. For instance, aspartate supplementation enables cells to proliferate under pharmacological inhibition of electron transport chain (ETC), suggesting that although adenosine triphosphate (ATP) production is the primary function of OXPHOS, proliferating cancer cells obtain oxidizing power and aspartate for pyrimidine biosynthesis through aerobic respiration^[14,15]^, thus, in addition to ATP or ROS levels, there may be other mechanisms underlying the antiproliferative effects of ETC inhibition. Several compounds block complex I (i.e., NADH: ubiquinone oxidoreductase), with high affinity viz. IACS-010759^[16]^, IM156^[17]^, BAY87-2243^[18]^, and ME-344^[19]^.

The OXPHOS inhibitor IACS-010759 is currently being evaluated in phase 1 clinical trials in relapsed/refractory AML [Figure 1]^[16]^. BAY87-2243, an inhibitor of hypoxia-inducible factor-1 (HIF-1) that inhibits mitochondrial complex I activity, causes grade III nausea/vomiting [NCT01297530], while ME-344 showed no clinical efficacy in patients with solid cancers^[18,20]^.

Oxidative phosphorylation integrity is maintained by a protease, caseinolytic protease proteolytic subunit (ClpP) in AML cells^[21]^. The altered function of this protease contributes to misfolding or degradation of respiratory chain subunits and their accumulation, causing respiratory chain dysfunction. The imipridones ONC201 and ONC212 inhibited the growth and viability of leukemia cells by hyperactivating mitochondrial ClpP and thus selectively proteolyzing certain mitochondrial matrix proteins^[21]^. A significant reduction in the leukemic burden and improved survival was observed in mice that were administered ONC201 orally^[21]^. In a clinical trial, a single oral dose of ONC201 resulted in a decrease in circulating blasts and a subsequent increase in platelet counts in patients with relapsed/refractory AML^[21]^.

Mubritinib (TAK-165) is a direct and ubiquinone-dependent ETC complex I inhibitor which induces ROS accumulation. Mubritinib also decreases ATP/ADP and NAD/NADH concentration ratios and inhibits the activities of PDH, tricarboxylic acid cycle (TCA), and OXPHOS, which results in the death of AML cells treated with mubritinib^[22]^. In vivo, mubritinib delayed AML development without affecting normal hematopoiesis in mice^[22]^. An isoquinoline alkaloid, berberine, targets mitochondrial ETC complex I, leading to reduced mitochondrial activity and enhanced antileukemic effects in vitro and in vivo when combined with IDH1 mutant inhibitor AG-120^[23]^.

A rationally designed lipoic acid analog, CPI-613, is currently being investigated in clinical trials for patients with pancreatic cancer and acute myeloid leukemia^[24]^. Mechanistically, CPI-613 inhibits the TCA cycle by displacing lipoic acid, a cofactor for the TCA cycle enzymes pyruvate dehydrogenase and α-ketoglutarate dehydrogenase. This displacement, in turn, prevents the enzymes’ activity and halts the TCA cycle^[25]^.

Metformin is being evaluated in combination with chemotherapy in different cancers; it sensitizes AML cells to Ara-C by reducing the mitochondrial transfer from bone marrow stromal cells (BMSCs) and OXPHOS in the recipient cells^[26]^. Metformin was found to synergistically sensitize AML cells to Ara-C by inhibiting the mammalian target of rapamycin (mTOR)C1/P70S6K pathway^[27]^. By promoting autophagy and apoptosis mediated by mTOR, metformin sensitizes FLT3-ITD-positive acute myeloid leukemia to sorafenib^[28]^. Additionally, metformin and phenformin are also potentiators of the cytotoxicity of Bcl2 antagonist - ABT-737 against leukemia^[29]^. By inhibiting protein production, metformin downregulates antiapoptotic Mcl-1 and Bcl-xl expression, resulting in a synergistic anti-tumor effect with ABT-199 in AML^[30]^. It also exhibited anticancer activity with the MCL1 inhibitor S63845 via redox remodeling^[31]^. IM156 is another oral biguanide OXPHOS inhibitor of mitochondrial ETC Protein Complex 1, which activates AMPK and is well tolerated at doses active in preclinical models with modest clinical efficacy [NCT03272256] in solid cancer patients^[32]^. Though the biguanides showed promising efficacy in preclinical studies, they are still under development and warrant further investigation in clinical trials for AML to establish their clinical utility.

### Role of mitochondrial OXPHOS in chemoresistance

Cytarabine remains the backbone of AML chemotherapy. Interestingly, pre-existing and persisting cytarabine-resistant cells do not necessarily reside in a quiescent or LSC compartment but rather have higher OXPHOS levels, with polarized mitochondria and an increased mitochondrial mass^[33]^. The ability of cytarabine to induce mitochondrial ROS is a major determinant of cytarabine sensitivity in primary AML cells. Resistance to cytarabine is often mediated by SIRT3, a NAD+-dependent protein deacetylase that deacetylates mitochondrial anti‐oxidant enzymes, including IDH2 and superoxide dismutase 2 (SOD2)^[34]^. SIRT3‐mediated deacetylation increases the enzymatic activity of its antioxidant targets, thereby restricting cytarabine-induced mitochondrial ROS production and resulting in reduced apoptosis and increased chemoresistance^[34]^. In addition, SIRT3 drives OXPHOS and increases the levels of both dihydronicotinamide adenine dinucleotide phosphate (NADPH) and reduced glutathione (GSH) while decreasing the concentrations of their respective oxidized forms. SIRT3 activation conferred resistance to chemotherapy *in-vivo* and its inhibition showed a synergistic effect with cytarabine^[34]^. SIRT3 SUMOylation was higher in Ara-C sensitive primary cells, but lower in resistant primary cells^[35]^. De-SUMOylation of SIRT3 is mediated by SUMO-specific peptidase 1 (SENP1), which enhances its deacetylase activity by inhibiting its protein degradation. When SIRT3 is de-SUMOylated, the expression of HES1, a negative regulator of FAO, is decreased and FAO is upregulated; either FAO inhibitors or overexpression of HES1 can attenuate this effect^[35]^. The use of Momordin-Ic a, SENP1 inhibitor or HES1 overexpression in vitro and in vivo showed synergy with cytarabine to eradicate AML cells^[35]^. Although these studies demonstrate a role for SIRT3 in chemoresistance in AML, the number of patients included in these studies is limited, and therefore validation is required in larger cohorts.

Notably, following cytarabine therapy, residual cells showed increased FAO, increased CD36 expression, (a fatty acid translocase) and higher expression of an *OXPHOS* gene signature that is predictive of clinical response to treatment in PDX models and AML patients^[33]^. Inhibition of mitochondrial protein biosynthesis, electron transfer, or FAO resulted in reduced OXPHOS and strikingly increased efficacy of cytarabine in AML^[33]^. Another mechanism of cytarabine resistance is mediated by upregulation of CD39, an ectonucleotidase that is localized on the cell surface and hydrolyses extracellular ATP and ADP to produce adenosine. Increased activity of CD39 promotes mitochondrial activity and biogenesis by activating the cAMP-mediated adaptive mitochondrial stress response, which promotes resistance to cytarabine. The activation of cAMP-PKA signaling driven by CD39 through P2RY13 purinergic receptor causes metabolic reprogramming and promotes the survival of chemo-resistant AML cells^[36]^. Thus, CD39-P2RY13-cAMP-OxPHOS axis plays a key role in cytarabine resistance; inhibiting CD39 ecto-ATPase activity enhanced cytarabine’s cytotoxicity in AML by blocking the mitochondrial reprogramming caused by the drug^[36]^.

## TARGETING IDH1/2M

The isocitrate dehydrogenases enzymes, IDH1 and IDH2, catalyze the oxidative decarboxylation of isocitrate to α-ketoglutarate (α-KG) in the cell cytoplasm and mitochondria, respectively, and reduce NADP+; this, in turn, contributes to the generation of NADPH. α-KG is essential for the optimal functioning of multiple metabolic and epigenetic processes, while NADPH functions as a reducing agent and plays a crucial role in maintaining the cellular redox state. IDH1/2 mutations have been found in 1 of every 5 AML patients with a higher preponderance among patients with normal karyotypes^[37,38]^. IDH1/2 mutations detected in AML are predominantly heterozygous point mutations that not only render the protein incapable of carrying out decarboxylation of isocitrate, but confer it the ability to produce 2-hydroxyglutarate (2-HG), an oncometabolite, by NADPH-dependent reduction of α-KG^[39]^. 2-HG inhibits several α-KG-dependent dioxygenases, such as demethylases and the TET family of 5-methlycytosine hydroxylases^[40]^. TET regulates the gene expression of its target genes through DNA demethylation and chromatin modification, such as H2B O-linked N-acetylglycosylation (O-GlcNAcylation) or H3K4 trimethylation^[41]^. The loss of α-KG in conjunction with the suppression of α-KG-dependent dioxygenases mediated by 2-HG promotes leukemogenesis^[42,43]^. In addition, loss of NADPH due to IDH mutations perturbs the regulation of redox balance in the cell^[44]^.

In recent years, ivosidenib (AG120), an IDH1 inhibitor, has been used in the treatment of relapsed and refractory AML^[45]^ while many other inhibitors including Olutasidenib (FT-2102), IDH305, Vorasidenib (AG881), BAY1436032, LY3410738 and DS-1001 are currently being evaluated in Phase I/II clinical trials in patients with IDH1/2 mutant AML. The IDH2 inhibitor Enasidenib (AG-221) and Ivosidenib are approved for relapsed/refractory AML in the presence of relevant mutations and ivosidenib was recently approved, in combination with hypomethylating agents, as first-line therapy for IDH1 mutant AML [Figure 1]. The IDH1 mutant AML cells carrying IDH1R132H, IDH1R132C, IDH1R132G, IDH1R132L and IDH1R132S mutations undergo myeloid differentiation when treated with BAY1436032, a novel pan mutant IDH1 inhibitor which specifically inhibits R-2HG production and colony growth in these cells. Aside from that, the compound affects DNA methylation and attenuates hypermethylation of histones. In two independent xenograft mouse models derived from AML patients with IDH1 mutation, treatment with BAY1436032 increased leukemic blast clearance, myeloid differentiation, and stem cell depletion while prolonging survival^[46]^. Combining azacitidine and BAY1436032 increased survival and depleted LSCs by inhibiting MAPK/ERK and RB/E2F signaling^[47]^. In a study conducted by Shih *et al*., AG-221 treatment reduced aberrant hypermethylation in several genes involved in hematopoietic differentiation, suggesting an epigenetically driven differentiation effect. As a single agent, AG221 or 5-azacitidine in vivo stimulated the production of mature myeloid cells from mutant leukemic progenitor blasts. Importantly, in Idh2^R140^QFlt3^ITD^ mutant AML combination therapy with FLT3 inhibitor AC220 (quizartinib), significantly decreased mutant cell burden and led to the recovery of normal hematopoiesis from non-mutant stem/progenitor cells^[48]^.

The emergence of second-site IDH2 mutations in trans, at glutamine 316 (Q316E) and isoleucine 319 (I319M), which are located at the interface of the enasidenib binding site in the IDH2 dimer, were found to confer therapeutic resistance to enasidenib. Although the expression of these mutant proteins alone is not sufficient for the synthesis of 2HG, the presence of R140Q mutation in trans produces 2HG, which is resistant to inhibition by enasidenib^[49]^. Mutations in the IDH dimer-interface in cis were found to confer resistance to ivosidenib in AML^[49]^. In a high-content shRNA screen of isogenic leukemia cells expressing wild-type and mutant IDH1, Chan *et al*. (2015) found that survival of IDH1/2 mutant cells was highly dependent upon BCL-2 and BCL-W expression^[50]^. Venetoclax-based therapy was effective in 6 out of 7 patients with IDH1 mutations who had previously been treated with ivosidenib; however, a lower response rate was reported for patients with FLT3-ITD mutations, indicating that venetoclax is an effective salvage therapy in patients who previously received IDH1/2 inhibitors^[51]^. IDH305 is a selective oral IDH1 inhibitor that specifically targets R132* IDH1 mutation. There is a phase I clinical trial currently being conducted on this drug for the treatment of advanced malignancies, including relapsed/refractory AML and myelodysplastic syndromes (MDS) [NCT02381886]^[52]^.

### Vulnerabilities in IDH mutated AML

Mutations in IDH1/2 have been reported to confer sensitivity to several chemotherapeutic agents in most cancer types, including colorectal cancer^[53]^, glioma^[54,55]^, cholangiocarcinoma^[53,56]^, and AML^[50,57]^. Conversely, inhibition of mutant IDH1/2 may counteract the cytotoxic effect of chemotherapeutic drugs^[58]^. In IDH1/2 mutants, D2HG mediates the downregulation of ATM, a DNA damage response gene. Therefore, IDH1/2 mutant AML cells are sensitive to DNA damage-causing agents, such as daunorubicin, and the PARP inhibitors olaparib and talazoparib, while IDH1/2 mutant inhibitors had a protective effect against these treatments^[58]^. Intriguingly, IDH1/2 mutation sensitized primary AML cells to ABT199 by inhibiting cytochrome c oxidase (i.e., Complex IV of ETC) by 2HG^[50]^. The cytochrome c oxidase complex contains two heme moieties (a and a3) and two copper atoms (CuA and CuB)^[59]^. A binding of (R)-2-HG at or near the binuclear center of heme a3 and CuB inhibits cytochrome c oxidase activity, thereby blocking oxygen reduction at that site^[50]^. Suppression of cytochrome c oxidase activity results in oxygen deprivation, which triggers activation of BAX/BAK and leads to outer membrane permeabilization and apoptosis. When BAX/BAK are bound to BCL-2, permeabilization is prevented, but ABT-199 disrupts this binding so that IDH1/2 mutant cells die, while wild-type IDH1/2 cells are relatively unaffected^[33]^. IDH1/2 mutant AML exhibited a superior response (36%) to BCL-2 inhibition compared to IDH1/2 wildtype (9%)^[60]^. Patients with IDH-mutant AML show robust responses to venetoclax + azacytidine^[61]^. This is a great example of a driver mutation that creates a unique metabolic vulnerability^[62]^.

Primary resistance to IDH inhibitors is primarily associated with leukemia stemness, while acquired resistance is often conferred by mutations in the genes belonging to the RUNX1/CEBPA or RAS-receptor tyrosine kinase (TK) pathways or genes such as BCOR, and TET2^[63]^. These mutations often affect transcription factors that are involved in hematopoietic and myeloid differentiation, particularly RUNX1 and CEBPA. An association was found between co-mutation(s) of RUNX1 at baseline and a lower CR rate with IDH inhibitor^[63]^. RUNX1 mutations were also the most frequently acquired mutations in relapsed disease. In addition, co-occurrence of CEBPA mutation was associated with a lack of response to IDH inhibitor, and the mutation was also reported to be acquired at relapse^[63]^. Because of their roles as differentiation factors, RUNX1 and CEBPA mutations may interfere with differentiation signals induced by IDH inhibitors, resulting in clinical resistance^[63]^. Almost 30% of relapse cases were found to have acquired mutations in either NRAS or KRAS during relapse^[63]^. Interestingly, Tateishi *et al*. found that IDH1 mutation may cause addiction to NAD+ in solid cancers^[64]^. The mutant form of IDH1 lowered NAD+ levels through downregulation of the enzyme nicotinate phosphoribosyltransferase (NAPRT1) of the NAD+ salvage pathway and thus sensitized cells to NAD+ depletion when combined with inhibition of nicotinamide phosphoribosyltransferase (NAMPT). Depletion of NAD+ activated AMPK, triggering pro-cell death autophagy, resulting in cell death^[64]^.

## TARGETING AMINO ACID METABOLISM

### Role of glutamine and glutaminolysis: as cellular building blocks and in ROS mitigation homeostasis

**Figure 2:**
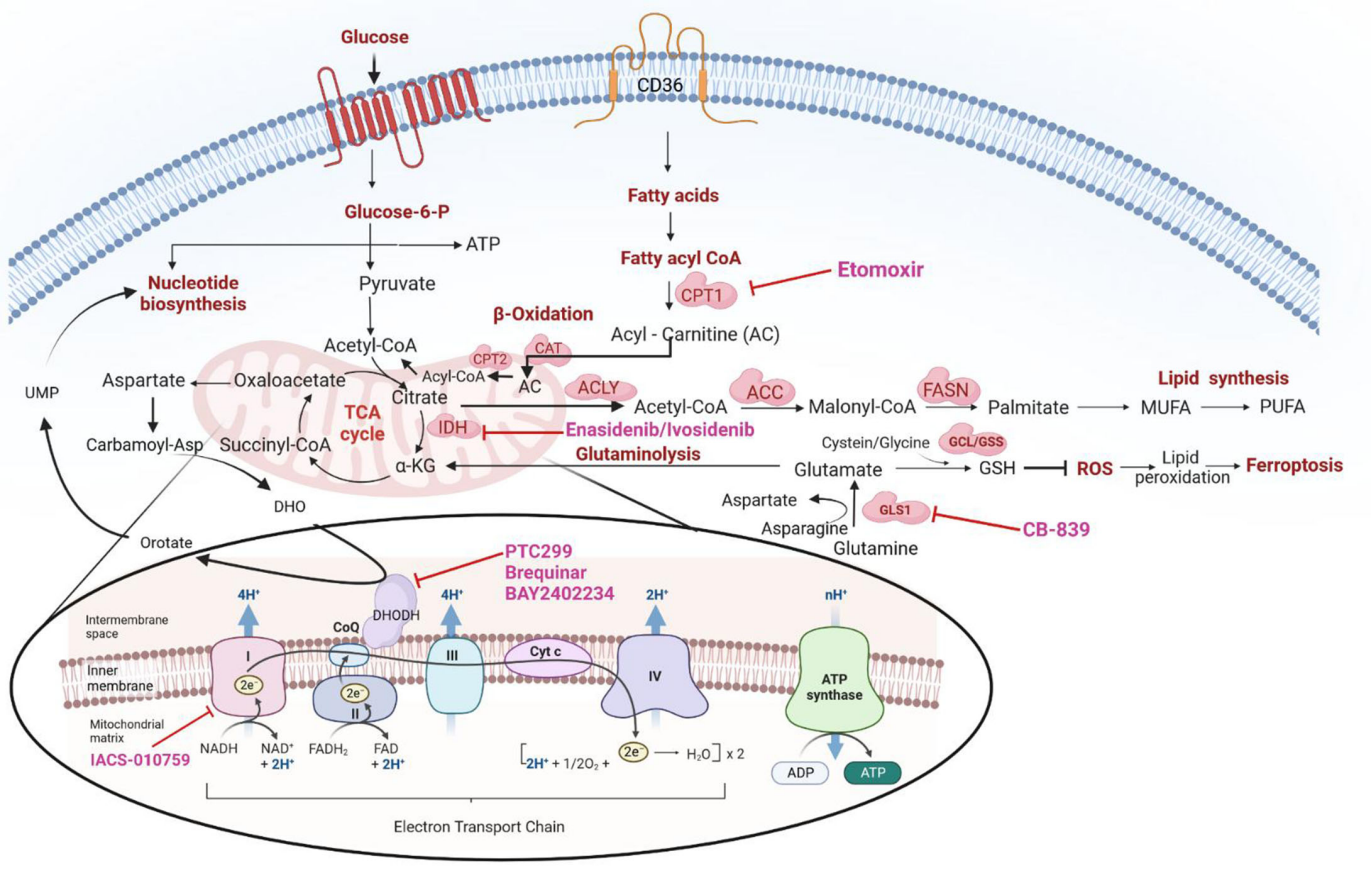
Overview of cellular metabolic pathways and their crosstalk -including: (1) glycolysis; (2) TCA cycle; (3) OXPHOS; (4) glutaminolysis; (5) nucleic acid biosynthesis; (6) lipid synthesis; (7) fatty acid import and oxidation; (8) ROS mitigation by GSH; (9) ferroptosis. ACC: Ac-CoA carboxylase; ACLY: ATP citrate lyase; ADP: adenosine diphosphate; ATP: denosine triphosphate; α-KG: α-ketoglutarate; CAT: carnitine acetyltransferase; CPT1: carnitine palmitoyltransferase 1; CPT2: carnitine palmitoyltransferase 2; DHO: dihydroorotate; DHODH: dihydroorotate dehydrogenase; FAD: flavin adenine dinucleotide; FASN: FA synthase; GSH: glutathione; IDH: isocitrate dehydrogenase enzyme; GCL: glutamate-cysteine ligase; GSS: glutathione synthetase; GLS1: glutaminase 1; NAD: nicotinamide adenine dinucleotide; NADH: nicotinamide adenine dinucleotide (NAD) + hydrogen (H); MUFA: monounsaturated fatty acid; OXPHOS: oxidative phosphorylation; PUFA: polyunsaturated fatty acid; ROS: reactive oxygen species; TCA: tricarboxylic acid cycle; UMP: uridine monophosphate.

Besides serving as substrates for protein biosynthesis, amino acids participate in the biosynthesis of purine and pyrimidine nucleotides and provide precursors for energy generation. For instance, acetyl-CoA can be generated by the ketogenic amino acids leucine and lysine. Similarly, the glycogenic amino acids alanine and glycine generate pyruvate and TCA cycle intermediates. Glutamine addiction has been reported in some primary AML patient samples and cell lines^[65-67]^, and can drive the TCA cycle by providing glutamate; through glutaminolysis, glutamate is then converted to α-ketoglutarate. In addition, it can serve as a substrate in the synthesis of GSH, a ROS mitigator^[66,68]^ [Figure 2]. Gregory *et al*. demonstrated that inhibition of glutaminase, an enzyme responsible for converting glutamine to glutamate, could perturb GSH balance and adversely affect the redox state in AML^[69]^. AML development in NSG mice was dramatically inhibited by knocking down the glutaminase gene GLS1, leading to the failure of conversion of glutamine to glutamate (glutaminolysis); this, in turn, induced apoptosis, and exhibited a synergistic effect in sensitizing leukemic cells to venetoclax^[66]^.

### Drivers of glutaminolysis

The cellular influx and efflux of glutamine in exchange for leucine influences the intracellular levels of glutamine, which is critical for mTORC1 activity, and translation and control of autophagy to coordinate cell survival and proliferation^[70]^. Solute-carrier family 1 member 5 (SLC1A5) is responsible for glutamine transport into the cells. Glutamine transport is essentially linked to leucine uptake. The bidirectional transporter, SLC7A5/3A2 is responsible for the uptake of leucine in exchange of glutamine^[71]^. Knockdown of SLC1A5 in a murine model of AML resulted in increased apoptosis and reduced tumor formation^[72]^.

Translation of cellular mRNAs is tightly regulated by mTOR. The mTOR pathway regulates the phosphorylation of 4E-BP1 (at serine 65), which is required for the initiation of translation^[73]^. Asparaginases are routinely used in the treatment of acute lymphoblastic leukemia ; they catalyze the conversion of asparagine and glutamine to aspartate and glutamate, respectively, and help to reduce the concentrations of asparagine and glutamine in the blood^[74]^. Glutamine depletion induced by L-asparaginases inhibits 4EBP1 phosphorylation at residue S65 and decreases protein synthesis in AML cell lines^[72]^. When 4EBP1 is dephosphorylated, it binds to eIF4E, resulting in inactivation of translational initiation complexes and a reduction in cap-dependent protein synthesis. In Complex karyotype AML, co-treatment with venetoclax and pegcrisantaspase, an asparaginase, significantly diminished cellular protein synthesis including that of MCL-1, an important antiapoptotic protein in AML, by promoting the interaction between eIF4E and 4EBP1 on the cap-binding complex and interfering with active cap-mRNA translation downstream of mTOR signaling^[75]^.

Glutaminolysis is a therapeutically targetable vulnerability in FLT3-ITD and other tyrosine kinase (TK) activating leukemias. A synthetic lethality screen using CRISPR/Cas9, metabolomics analyses, and gene expression analysis reveals that FLT3-ITD cells develop glutamine metabolism dependence following FLT3 TK inhibition. FLT3 TK inhibition hinders glucose uptake and use, suppressing the enhanced central carbon metabolism that is found in FLT3-ITD cells and reversing the glycolytic phenotype; as a result, FLT3-ITD cells develop a metabolic dependency on glutamine metabolism. Despite a decrease in glucose uptake, TCA cycle activity and respiratory function are less affected by FLT3 TK inhibition; instead, they are supported by the continuous uptake of glutamine. FLT3 TK inhibition with concomitant suppression of glutamine metabolism using either GLS chemical inhibition or gene silencing, increased cell death in FLT3-ITD cells^[76]^. Glutaminolysis is also upregulated by FLT3-ITD through Foxo3a inhibition. Foxo3a activates the expression of MAX-interacting protein 1 (MXI-1), a protein which can block MAX from binding to Myc. This disruption of the interaction between Myc and MAX reduces the transactivation capacity of Myc^[77]^. In AML and other cancer cells, FLT3-ITD inhibits Foxo3a^[77,78]^, leading to Myc activation and glutaminolysis stimulation, which, in turn, generates anaplerotic substrates that fuel the TCA cycle, increasing citrate synthesis and its transport to cytoplasm. Increased citrate synthesis provides acetyl-CoA, which can feed into the biosynthesis of lipids and participate in the post-translational modification of proteins^[44]^.

### Agents targeting glutaminolysis

Glutaminolysis was recently identified as a therapeutic target for hematological and solid malignancies, and potent inhibitors are now being evaluated in clinical trials [NCT02071862 and NCT02071927]. Inhibition of glutaminolysis with CB-839 blocks glutamine utilization, resulting in decreased levels of glutamate, aspartate and several TCA cycle intermediates. As described in a previous study^[5]^, inhibition of glutaminolysis with BPTES in mutant IDH1/2 AML may be beneficial, as it arrested cell proliferation, decreased 2-HG levels and enhanced differentiation^[67]^. Interestingly, in AML harboring FLT3-ITD mutations, glutaminolysis inhibition alone confers only minor antiproliferative effects. Glutaminolysis inhibition is more effective when combined with FLT3-ITD tyrosine kinase inhibition^[76]^, which perturbs glycolysis and glucose utilization, with less effect on glutamine metabolism. Therefore, even though glycolysis and glucose utilization are impaired following FLT3 inhibition, glutamine can drive the TCA cycle and participate in GSH synthesis^[76]^. Notably, in AML cells treated with quizartinib, glutamine starvation resulted in the depletion of GSH and elevation of intracellular ROS. In addition to glutamine starvation, pharmacological inhibition using CB839 or glutaminolysis gene silencing enhanced the efficacy of FLT3-ITD tyrosine kinase inhibition by reducing the availability of intracellular glutamine (due to TCA cycle inhibition), and GSH generation, thus impacting redox metabolism^[76]^.

LSCs exhibit increased uptake and catabolism of amino acids. Venetoclax + azacytidine is highly effective in AML patients because of its action at inhibiting amino acid metabolism in LSCs^[79]^. Jones *et al*. demonstrated that depletion of amino acids, especially cysteine, was strongly associated with response to venetoclax + azacytidine regimen compared to ROS induction. LSCs in patients with de novo AML rely on amino acid metabolism to drive OXPHOS. In LSCs, the depletion of cysteine causes a reduction in GSH levels, which, in turn, leads to decreased glutathionylation of succinate dehydrogenase A (SDHA), a component of ETC complex II. This decreased glutathionylation of SDHA results in OXPHOS inhibition and ATP depletion, ultimately leading to LSC death^[80]^. BCL-2 inhibition is believed to promote the generation of mitochondrial permeability transition pores, resulting in leakage of mitochondrial membrane and reduced amino acid metabolism^[81]^. The combination of venetoclax and azacytidine has shown good efficacy in older AML patients, who are generally considered unfit for traditional chemotherapy due to high toxicity and poor outcomes with chemotherapeutic agents. In fact, in a Phase III study, this regimen resulted in higher complete remission compared to the placebo control (36.7% *vs*. 17.9%; *P* < 0.001), the composite complete remission was reported in 66.4% AML patients receiving ven + aza *vs*. 28.3% in placebo control; *P* < 0.001^[61]^. The study reported that almost half of all patients receiving azacitidine plus venetoclax had a first response (complete remission or complete remission with incomplete hematologic recovery) before starting cycle 2, and their remissions lasted an average of 17.5 months. Of note, the remission rate after venetoclax + azacytidine in patients with *de novo* AML is independent of the genetic background, suggesting that venetoclax targets a unique metabolic program of LSCs^[61]^.

### Targeting arginine

Primary AML cells are auxotrophic for glutamine, cysteine, and arginine^[80,82]^. As they lack argininosuccinate synthetase-1 (ASS1), a key enzyme required for the generation of arginine, these cells rely on arginine import^[83]^. The depletion of extracellular arginine via the use of pegylated arginine deiminase, which converts plasma arginine to citrulline, induced responses in AML through the activation of caspases^[83]^. Depleting extracellular arginine shows synergy with cytarabine and enhances its cytotoxicity^[82]^. Arginine and cysteine metabolism are also controlled by the stroma in CLL, which may promote the resistance and growth of leukemic cells^[84]^; this can be targeted by arginine depletion.

### Targeting branched-chain amino acids

The survival of HSCs and LSCs is influenced by branched-chain amino acids (BCAAs), such as leucine, isoleucine, and valine. For instance, valine is essential for survival and maintenance of HSCs^[85,86]^. BCAAs promote cancer growth in *de novo* AML^[87]^. LSCs exhibit elevated levels of branched-chain amino acid transaminase 1 (BCAT1)^[88]^, a cytoplasmic aminotransferase that generates glutamate by transferring an amino group from a BCAA to α-KG^[89]^, which can in turn fuel OXPHOS.

## TARGETING PYRIMIDINE BIOSYNTHESIS

Proliferating cells require an abundance of nucleotides. They heavily depend on *de novo* nucleotide biosynthesis to meet their nucleotide requirements. As expected, the expression of enzymes involved in nucleotide biosynthesis is elevated in cancer. Uridine monophosphate (UMP) is generated in *de novo* nucleotide biosynthesis from glutamine. Similarly, dihydroorotate dehydrogenase (DHODH) is a mitochondrial enzyme that converts dihydroorotate (DHO) into orotate through oxidation, in *de novo* pyrimidine biosynthesis and donates the electrons to ubiquinone to generate ubiquinol, which is then re-oxidized by ETC complex III^[90]^. In experimental models of AML, inhibition of DHODH enhances myeloid differentiation^[90]^. In addition, a decrease in leukemia-initiating cells, as well as increased survival in vivo, have also been observed following the inhibition of DHODH^[91]^. DHODH inhibition promotes myeloid differentiation by depleting uridine, a precursor of uridine diphosphate-N-acetylglucosamine (UDP-GlcNAc). The depletion of uridine diphosphate-GlcNAc decreases O-linked N-acetylglycosylation (O-GlcNAc) of several proteins including Akt, TET family of proteins and c-Myc^[91]^. The loss of O-GlcNAc modification following DHODH inhibition reduced the stability of c-Myc and thereby promoted differentiation in AML^[92]^. In addition, DHODH inhibition revealed the dysregulation of several key genes that are involved in a maturation block of AML cells, such as HOXA9, FLT3 and c-Myc^[93]^. Of note, DHODH inhibition using BAY 2402234 induced acute downregulation of HMGA1, a chromatin remodeler that is implicated in stemness, and would otherwise negatively regulate differentiation^[93]^. In clear cell renal cell carcinoma, the DHODH inhibitor selectively inhibited tumor growth in GPX4-low tumors, while combined treatment with sulfasalazine, which also induces ferroptosis, synergistically inhibited the growth of GPX4-high tumors^[94]^.

Notably, several clinical trials have reported that brequinar, a DHODH inhibitor, has limited efficacy, likely related to a schedule that did not allow sustained exposure^[91,95]^. ETC complex III helps in maintaining the oxidation of ubiquinol, which is essential to supporting DHODH activity^[96]^. Interventions targeting respiratory Complex III would increase the generation of ROS and block the activity of DHODH and pyrimidine biosynthesis^[97,98]^, and could thus be an effective two-pronged strategy involving the exploitation of cell death promoted by ROS^[99]^ and differentiation induced by DHODH inhibition. Alternative oxidase (AOX) can restore DHODH activity and reactivate CoQ redox-cycling following DHODH inhibition^[100]^.

Aspartate biosynthesis is a crucial step in pyrimidine biosynthesis, which requires ETC complex I activity^[15]^. AML’s dependency on ETC complex I has been well-documented. As a result, there has been an increasing interest in targeting ETC complex I and induction chemotherapy together.

## TARGETING NICOTINAMIDE METABOLISM

LSCs from relapsed/refractory AML patients are resistant to venetoclax + azacytidine, in contrast to those from *de novo* AML patients, suggesting that they have alternative resistance mechanisms. Jones *et al.* (2020) determined whether LSCs from relapsed/refractory AML patients exhibited altered metabolome profiles compared to those from *de novo* AML; they identified 18 metabolites that were upregulated, including amino acids and nicotinamide^[101]^. Interestingly, relapsed LSCs exhibited an enhanced rate of NAD+ synthesis driven by the salvage pathway, with no difference in the protein levels of NAMPT, the rate-limiting enzyme responsible for the generation of NAD+ from nicotinamide, suggesting an increase in nicotinamide uptake in relapsed/refractory LSCs^[101]^. The elevation of nicotinamide metabolism in relapsed LSCs, activates amino acid metabolism and FAO, which in turn drives OXPHOS. An increase in OXPHOS would help LSCs to confer resistance to venetoclax + azacytidine^[101]^.

Unlike *de novo* LSCs, relapsed/refractory LSCs exhibit extensive metabolic plasticity, which enables them to adapt alternative mechanisms for activating OXPHOS, a key mechanism for developing resistance to chemotherapy^[101]^. Given that therapeutic options are limited for relapsed/refractory AML, the inhibition of NAMPT, the rate-limiting enzyme for NAD+ synthesis, may be an effective approach^[101]^. Notably, relapsed/refractory LSCs were selectively eradicated by the genetic and pharmacological inhibition of NAMPT, while normal hematopoietic stem/progenitor cells were spared. Of note, in secondary transplantation of relapsed/refractory AML LSCs, in-vivo treatment with APO866, a small molecule inhibitor of NAMPT, compromised the engraftment^[101]^.

## TARGETING FATTY ACID METABOLISM

FA catabolism has been implicated in regulating HSC fate (i.e., maintenance *vs*. exhaustion) by providing the required energy to allow the proper execution of asymmetric division, favoring the maintenance and function of the HSC compartment. Loss of the mitochondrial FAO activator peroxisome proliferator-activated receptor δ (PPAR-δ) perturbs HSC maintenance, whereas treatment with PPAR-δ agonists restores HSC maintenance by promoting asymmetric HSC division^[102]^. PPAR-δ activates FAO in mitochondria. LSCs from *de novo* AML patients drive OXPHOS exclusively using amino acid metabolism. In contrast, LSCs from relapsed/refractory AML patients tend to compensate significantly through increased FA metabolism^[79]^. Despite the importance of FAs for mitochondrial metabolism in LSCs, aberrant lipid metabolism may also promote cancer cell proliferation by increasing its energy availability, structural lipid-based building blocks, and signaling molecules^[103]^. Vriens *et al.* demonstrated that cancer cells exhibit extensive plasticity in FA metabolism and can facilitate membrane biosynthesis during proliferation through the FA desaturation pathway^[103]^. Further investigation is needed to determine whether such metabolic plasticity exists in leukemic cells and if it could be targeted.

Mcl1 is an antiapoptotic protein that is known to interact with proapoptotic proteins in mitochondria. Mcl1 upregulation is common in venetoclax-resistant AML^[104]^. Interestingly, MCl1 in cancer cells regulates lipid metabolism through a functional gain. This is achieved by binding its α- helix of the BH3 domain to very long-chain acyl-CoA dehydrogenase (VLCAD), a key enzyme of the mitochondrial FAO pathway, through its hydrophobic surface^[105]^. Thus, MCL1 not only suppresses apoptosis in cancer cells, but also regulates β-oxidation in response to stress. Of note, Mcl-1 is essential for the efficient assembly of F1F0-ATP synthase oligomers and thus is important for maintaining mitochondrial cristae structure and efficient ATP synthesis^[106]^. Another study revealed the bifunctional activities of BAD, demonstrating glucokinase regulation by the proapoptotic BH3 domain of BAD^[107]^. The findings of these studies have opened a new paradigm, revealing the role of Bcl2 family proteins in not only regulating mitochondrial apoptosis but also energy metabolism in cancer cells. It would be interesting to further explore whether Bcl2 family protein mediated regulation of metabolism plays a role in resistance to standard chemotherapies in AML and to further identify downstream druggable non-canonical targets.

### Fatty acid metabolism and resistance to therapy

Cellular levels of ceramide are key determinants of response to several chemotherapeutic drugs. Modulation of the ceramide pathway has been implicated in apoptosis induced by daunorubicin in AML cells^[108]^. Ceramidases convert ceramide to sphingosine. Kao *et al*. found that daunorubicin and cytarabine treatment resulted in the increased expression and activity of enzymes involved in the ceramide pathway, leading to a decrease in ceramide levels and an increase in ceramide 1- and sphingosine 1-phosphate levels. These perturbations induced remodeling of mitochondria in cancer cells and promoted chemoresistance^[109]^. For instance, FLT3-ITD signaling activation suppresses pro-cell death lipid ceramide generation in AML cells^[110]^. Intriguingly, LCL-461, a mitochondria-targeted ceramide analog, effectively induced lethal mitophagy in human AML blasts and crenolanib (FLT3-ITD inhibitor)-resistant AML xenografts, suggesting that activation of mitochondrial ceramide synthesis overcomes resistance to FLT3 inhibition^[110]^.

A previous study reported that *de novo* cholesterol synthesis through the mevalonate pathway plays a key role in AML^[111]^. Recently, using genome-scale metabolic modeling, Karakitsou *et al*. demonstrated that AraC-resistant cells could be targeted by suppressing cholesterol biosynthesis through the inhibition of squalene synthase^[112]^.

Acetyl-CoA is converted to malonyl-CoA by Ac-CoA carboxylases (ACCs). FA synthase (FASN) catalyzes the condensation of malonyl-CoA and Ac-CoA to generate palmitate as the first product of FA synthesis^[113]^. FASN is significantly elevated in AML blasts compared to healthy granulocytes or CD34+ hematopoietic progenitors at the RNA level. Inhibition of FASN expression on one side accelerated differentiation of APL cells, on the other hand, sensitized ATRA resistant -non-APL AML cells to ATRA, by promoting translocation of transcription factor EB (TFEB) to nucleus and lysosomal biogenesis^[114]^.

Cellular stress directly regulates FA metabolism by affecting the enzymatic activity of Acetyl-CoA carboxylases **(**ACC), namely ACC1 and ACC2. AMPK inhibits ACC under energy stress and thus activates fat synthesis and catabolism. Nutrient abundance, on the other hand, downregulates the activity of AMPK, and ACC1 and ACC2 are no longer repressed^[115]^. This suggests that ACCs act as a regulatory node in FA metabolism by sensing nutrient availability and balancing the switch between anabolic and catabolic processes. Of note, ACC1 suppressed growth-promoting activity and promoted the generation of ROS in primary BM cultures. In addition, ACC1 has been shown to promote myeloid differentiation and delay the development of AML in mice^[116]^. Hydroxylation of ACC2 by prolyl-hydroxylase 3 (PHD3) increases the activity of ACC2, which, in turn, prevents FA utilization^[117,118]^. PHD3 levels are decreased in AML^[119]^, thus generating a vulnerability due to dependence on FAs, which can make FAO inhibition an attractive strategy to control AML.

Venetoclax + azacytidine-resistant LSCs exhibit increased levels of several metabolites that are responsible for the transport of FAs into mitochondria at baseline, including acetyl-carnitine, iso-butylryl carnitine, and hexanoyl-carnitine, compared to sensitive LSCs^[120]^. Venetoclax + azacydine treatment results in a reduction in amino acid uptake by LSCs. Enhanced FA transport facilitates FAO and allows LSCs to compensate for amino acid uptake loss caused by venetoclax + azacytidine treatment, resulting in resistance. However, this resistance can be overcome by the knockdown of very long-chain acyl-CoA dehydrogenase (ACADVL), an enzyme involved in mitochondrial FA beta-oxidation^[120]^, suggesting that FA beta-oxidation plays an important role in conferring resistance to venetoclax + azacytidine treatment in LSCs.

FAs are conjugated to carnitine by CPT1 to facilitate mitochondrial translocation, and both the PPARs and the coactivator-1 of PPARs modulate CPT1A expression^[121]^. Pharmacological inhibition of CPT1 by etomoxir depleted refractory LSC function^[122]^, and eliminated venetoclax+ azacytidine-resistant LSCs^[85]^. In addition, CPT1A inhibition by etomoxir induced cell death in AML cells and increased cells’ sensitivity to cytarabine^[123]^. CD36+ LSCs are characterized by an enhanced FAO rate, and disruption of mitochondrial metabolism by targeting CD36-FAO-OXPHOS drives leukemia cells to low OXPHOS and enhances AraC sensitivity^[19]^. In addition, topoisomerase inhibitor (mitoxantrone)-resistant AML cells showed activation of lipid metabolic pathways. Inhibition of FAO, using etomoxir in these cells, reduced colony formation ability, suggesting that FA metabolism and dependency on OXPHOS are key vulnerabilities, conferring chemoresistance in AML^[124]^.

## TUMOR MICROENVIRONMENT-MEDIATED METABOLIC PLASTICITY

### Tumor microenvironment-mediated OXPHOS regulation

BMSCs, which are composed of endothelial cells, osteoclasts, osteoblasts, adipocytes, and fibroblasts, form the BM microenvironment. BMSCs aid the survival of AML cells and mediate resistance to therapy. Cell-cell communications in the BM microenvironment play a key role in aiding AML cell survival, disease progression, and therapeutic response through the transfer of biomolecules or cellular organelles, such as mitochondria^[125]^. Of note, BMSCs supply mitochondria to AML cells in vitro, thus providing them with additional energy^[126]^. AML cells were observed to enhance their mitochondrial mass up to 14% by mitochondrial uptake from BMSCs; this transfer increased in response to chemotherapy and contributed to resistance by reducing mitochondrial depolarization^[126]^. AML cells exhibited up to a 1.5-fold increase in mitochondrial ATP production following the uptake of mitochondria from BMSCs, were less prone to depolarization of mitochondria following chemotherapy, and showed improved survival^[126]^. Of note, transfer of mitochondria to AML cells from BMSCs in the presence of chemotherapeutic agents occurs predominantly through endocytosis and requires cell-cell contacts^[12]^. The NADPH oxidase, NOX2 generates superoxide in AML cells, which promotes mitochondrial transfer to AML blasts from BMSCs through AML-derived tunneling nanotubes. Interestingly, suppression of NOX2 reduced the transfer of mitochondria to AML blasts and resulted in enhanced cell death and improved survival of AML-bearing mice^[126]^. AML cells show high OXPHOS levels when cocultured with BMSCs. Hou *et al*. showed that the elevation of OXPHOS levels in AML cells in coculture was dependent on the activation of mitochondrial serine-phosphorylated STAT3 (pS-STAT3)^[127]^. AML cells in coculture induced the secretion of interleukin-6 from BMSCs, which in turn promoted the activation of total and mitochondrial STAT3 in AML cells, resulting in increased proliferation and chemoresistance^[127]^.

AML cells are known to harbor abnormally high levels of ROS. Mesenchymal stem cells, a component of BMSCs, support the survival of AML cells by influencing several key metabolic pathways and eliminating ROS. BMSCs expressing the intermediate filament protein nestin provide HSC niche function^[128]^. Forte *et al*. reported that after AML induction, unlike bulk stroma, the nestin-expressing BMSC cell population remains stable in MLL-AF9-driven AML, with no reduction in their number over time^[129]^. However, these cells undergo changes in their functional behavior to enhance the survival of MLL-AF9 AML cells and promote chemoresistance by promoting TCA cycle and OXPHOS. They also provide a GSH-mediated antioxidant defense, which is critical for ROS mitigation during leukemogenesis and chemotherapy^[129]^. Forte *et al*. observed a delay in leukemogenesis, following the depletion of Nestin^+^ BMSCs in primary AML mice^[129]^. Most importantly, the depletion of Nestin+ BMSCs in chimeric mice reduces AML, but not normal cells^[129]^. Thus, BMSCs help AML cells switch their energy sources and provide antioxidant defense mechanisms to survive chemotherapy.

### Tumor microenvironment mediated regulation of amino acid and pyrimidine metabolism

Induction chemotherapy causes transient stress in BM, which results in the elevation of glutamine, glutamate, and aspartate with a concomitant increase in the expression of several glutamine transporters, such as Slc1a5, Slc38a1, Slc7a5, and Slc7a6, but no change in the enzymes that metabolize glutamine or glutamate, in residual AML cells^[130,131]^. This suggests that glutamine metabolism in AML cells could be regulated by the tumor microenvironment^[130,131]^. Interestingly, van Gastel *et al*. showed that glutamine metabolism differed between AML and stromal BM cells, especially LepR+ mesenchymal stromal cells^[131]^. AML cells took up glutamine to maintain GSH but not to promote the TCA cycle. In contrast, in LepR+ mesenchymal stromal cells, glutamine entered the TCA cycle to produce aspartate^[131]^. Aspartate is transported to and supports pyrimidine biosynthesis in persistent MLL-AF9 AML cells, thus preventing the metabolic collapse triggered by chemotherapy^[130,131]^. These findings suggest that the tumor microenvironment plays a major role in chemoresistance, independent of cell-intrinsic signaling/apoptosis alterations. However, it is unclear whether aspartate-rich locations in the BM provide a protective niche, and if specific AML subclones compete for these spots. Further niche-specific studies are warranted to delineate whether mutational subclones compete for specific hotspots.

### Tumor microenvironment mediated regulation of fatty acid metabolism

Accumulating evidence supports the existence of metabolic symbiosis and bidirectional dialogues between leukemic cells and the BM adipose tissue niche, which provides diverse sources of lipids, such as FAs, cholesterol, and eicosanoids, to resident leukemic cells. Leukemic cells meet their energy needs by using FAs to fuel β-oxidation, which eventually supports anabolism^[33,132]^. When FAO was inhibited pharmacologically with etomoxir or ranolazine, AML cells were sensitized to apoptosis induction by ABT-737, and proliferation was inhibited in the setting of coculture with BMSCs^[123]^. Interestingly, a recent report described two forms of LSCs, distinguished by differences in their expression of CD36 and in their metabolism and cell cycle status. LSCs expressing CD36 (CD36+ LSCs) are often localized to gonadal adipose tissue (GAT) which is rich in fatty acids, indicating a tropism rich for microenvironments rich in FAs^[132]^. Specifically, these GAT-resident LSCs are more resistant to conventional chemotherapy because they exist in a quiescent cell cycle state. In the context of these findings, leukemic cells co-opt adipose tissue to create a microenvironment to support leukemia growth and provide a drug-resistant niche^[132]^. Further, leukemic burden in GAT for the MLL-AF9 model was considerably lower than that in BM, indicating that localization to GAT may be dependent on the specific oncogenes that drive the malignant transformation process^[132]^. An interesting question for future investigations would be to understand the role of FA metabolism in conferring drug resistance to LSCs in a niche-specific BM microenvironment.

## ROLE OF EXTRACELLULAR VESICLES IN CONFERRING RESISTANCE BY MEDIATING COMMUNICATION BETWEEN LEUKEMIC CELLS AND MICROENVIRONMENT TO ALTER METABOLISM

AML cells alter the BM by releasing exosomes, which promote the proliferation and survival of leukemic cells and suppress normal hematopoiesis. In a study by Javidi-Sharifi *et al*., the expression of FGF2 and its receptor, FGFR1, were both increased in several stromal cell lines and primary AML stroma. This heightened FGF2/FGFR1 signaling augmented greater secretion of exosomes^[133]^. Huang *et al*. (2021), studied the impact of inhibition of small extracellular vesicle (SEV) secretion from various sources on the progression of MLL-AF9 induced AML, as well as normal hematopoiesis^[134]^. A significant delay in AML progression occurred when SEV secretion from endothelial cells was inhibited, but not that from perivascular cells, megakaryocytes, or spleen stromal cells^[134]^. SEVs derived from endothelial cells contained high levels of Angiopoietin-like protein 2 (ANGPTL2) protein, which binds to the leukocyte immunoglobulin-like receptor B2 (LILRB2 receptor) and accelerates leukemia progression^[134]^. Moreover, Vacuolar protein sorting-associated protein 33B (VPS33B) governed the release of ANGPTL2-SEVs from endothelial cells and this release of ANGPTL2-SEVs was required for primary human AML cell maintenance. These findings demonstrate that SEVs have a role in cancer development and suggest that targeting the release of ANGPTL2-SEVs from endothelial cells would be an effective strategy in the treatment of some types of AML^[134]^. While IO-108, an antibody targeting LILRB2, is in Phase 1 clinical development for solid tumors^[135]^; LILRB4 antibody (IO-202) is in phase 1 cohort expansion clinical trial [NCT0437243] in combination with azacitidine and venetoclax, for the treatment of AML and likely to be effective in monocytic AML^[136,137]^.

Cytarabine and decitabine treatment resulted in significantly higher intracellular levels of cholesterol and HMGCR (3-hydroxy-3-methyl-glutaryl-coenzyme A reductase), the rate-limiting enzyme in the cholesterol synthesizing mevalonate pathway, in cultured AML and non-malignant cells. Concomitantly, these cells produced higher levels of SEVs^[138]^. Interestingly, there was an increase in HMGCR levels in SEVs in response to chemotherapy. These HMGCR+ SEVs promoted the growth of AML cells by upregulating intracellular cholesterol levels. This chemotherapy-induced enhancement of SEV secretion in AML cells was prevented by HMGCR inhibition^[138]^. Thus, HMGCR blockade could potentially provide a therapeutic alternative in AML by inhibiting cholesterol-driven chemoresistance caused by SEV signaling^[138]^. Another study reported the secretion of exosomes containing vascular endothelial growth factor (VEGF) and VEGF receptor (VEGFR) by AML cells. These exosomes induced glycolysis in human umbilical vein endothelial cells (HUVECs), which resulted in vascular remodeling and chemoresistance^[139]^.

BM stromal cells exposed to AML-derived exosomes exhibit decreased expression of genes supporting normal hematopoiesis (CXCL12, KITL, IL-7, IGF1) and osteogenesis (OCN, Col1A1, IGF1) and increased expression of genes supporting AML growth (DKK1, IL-6, CCL3)^[140]^. When BM is preconditioned with AML-derived exosomes, AML cell engraftment and growth are significantly accelerated, whereas disruption of exosome secretion in AML cells significantly reduced these cells’ ability to engraft and grow *in vivo*^[140]^*.* In coculture, serum and leukemic cells from patients with NPM1-mutated AML impair CD8+ T cells’ immune function^[141]^. Through SEVs, leukemic cells secrete miR-19a-3p into the tumor microenvironment. This process was mediated by the NPM1-mutated protein/CCCTC-binding factor (CTCF)/poly (A)-binding protein cytoplasmic 1 (PABPC1). MiR-19a-3p released from SEV was found to be internalized by CD8+ T cells, where it inhibited creatine import by repressing the expression of a transporter solute-carrier family 6 member 8 (SLC6A8)^[141]^. A reduction in creatine levels lowered ATP production and impaired CD8+ T cell immune function, which caused leukemic cells to escape the immune system^[141]^. Interestingly, the exosomes generated by fully engrafted AML PDX mice were similar to exosomes isolated from the plasma of the patients who had donated the cells for engraftment. These exosomes carried human proteins and leukemia-associated antigens, confirming their origin from AML patients^[142]^.

AML utilizes SEV miRNAs to target the mTOR pathway which suppresses protein synthesis in the LT‐HSC to elicit their quiescence^[143]^. While LT-HSCs are functional after the recovery, their DNA is damaged for a long time. Hematopoietic stem and progenitor cells (HSPCs) are generally susceptible to SEV entry and use the mTOR pathway, but HSCs selectively enter quiescence because of their unique sensitivity to protein synthesis disruption^[143]^.

## TECHNIQUES TO ASSESS MITOCHONDRIAL FUNCTION AND METABOLISM

A tightly coordinated flux of metabolites occurs in mitochondria, integrating bioenergetics, redox homeostasis, and anabolic metabolism. By assessing mitochondrial function, researchers have gained a better understanding of metabolism in cellular physiology, disease pathology, and etiology. The reduction of oxygen into water is essential to oxidative phosphorylation and measuring oxygen consumption is therefore one way to assess mitochondrial function^[144]^. The mitostress test using a high-throughput real-time mode approach in Agilent Seahorse XF analyzers is the best assay for measuring basal cell respiratory rate, ATP production, proton leak, maximum respiration, and spare respiratory capacity in an intact cell^[145]^. This single assay yields a wide range of parameters, providing insight into mitochondrial dysfunction and reveals the functional differences between cell types, cellular metabolic state (quiescent versus active), effect of drug candidates, impact of genetic modifications, or biochemical interactions^[146]^.

Mitochondria are known to generate approximately 90% of cellular reactive oxygen species (ROS)^[147]^. As a result of excessive production of ROS and/or decreased antioxidant defense activity, mitochondrial reactive oxygen species (mtROS) accumulate, causing oxidative stress (OS). It leads to oxidative damage that affects several cellular components, including lipids, DNA, and proteins^[147]^. To detect mitochondrial superoxide selectively, MitoSOX Red and Green are used, which are fluorogenic reagents designed for highly selective detection of superoxide in the mitochondria of viable cells^[148]^. A notable feature of these probes is that they are readily and specifically oxidized by superoxide alone and not by other ROS or reactive nitrogen species (RNS) generating systems at optimum concentration. However, the user should be aware of the cytotoxic or mitotoxic effect of these probes and their potential impact on mitochondrial morphology at higher concentrations^[149]^. Furthermore, oxidation of these probes is inhibited by superoxide dismutase. Small, cell-permeant dyes such as TMRM (tetramethylrhodamine, methyl ester) and TMRE (tetramethylrhodamine, ethyl ester) accumulate in active mitochondria and may be used to quantify mitochondrial membrane potential^[150]^. They appear bright in healthy cells with functioning mitochondria, but are dimmed or undetectable upon loss of mitochondrial membrane potential^[151]^.

Metabolome consists of all the molecules with low molecular weight (metabolites) within a cell, tissue or organism. Analyzing the metabolites within a cell, tissue or organism following a genetic change or physiological stimulus is known as metabolomics^[152]^. The metabolomics approach can be divided into two distinct types: untargeted metabolomics and targeted metabolomics. Targeted approaches focus on identifying and quantifying a small number of known metabolites, such as those found in clinical analyses. In untargeted approaches or hypothesis-generating approaches, data are obtained for as many species as possible, metabolites are annotated, and metabolic changes are reviewed regardless of whether they are known or unknown^[153]^. However, measuring metabolite concentrations by metabolomics only tells half the story. The accumulation of metabolites can be caused not only by increased production, but also by decreased consumption. The metabolic flux, which can be quantified as material flow per unit time, is equally important for understanding pathway activity^[153]^.

Unlike metabolites, fluxes are not physical entities that can be detected by mass spectrometry. But they can be inferred by using isotope tracers. Radioactive tracer studies laid the groundwork for modern metabolism research^[154]^. Today, similar studies can be performed using stable non-radioactive isotope tracers, which can be tracked quantitatively and broadly using MS or NMR^[155]^. Furthermore, an approach called metabolic activity screening integrates the metabolomics data with metabolic pathways and systems biology data, including proteomics and transcriptomics, in order to identify endogenous metabolites that may alter phenotypic characteristics and functionality^[156]^.

## CONCLUDING REMARKS

Nine therapeutic agents for AML have been developed and approved since 2017. Treatment improvements have increased the overall survival rate of AML patients, from 13% to 55% in those aged younger than 60 years and 8% to 17% in patients older than 60 years^[157]^. Further improvements in our understanding of the disease would further improve outcomes. The genetic makeup of AML, as well as its metabolic requirements for survival and proliferation, are heterogeneous. Diverse types of metabolic vulnerabilities, including dependence on OXPHOS, amino acid metabolism, nucleotide metabolism, and fatty acid metabolism, were reported. In addition, metabolic changes occurring in the tumor microenvironment have also been used as therapeutic targets. However, a thorough understanding of biomarkers of response to these therapies and mechanisms of resistance and combinatorial therapies would pave the way for better clinical management of AML. Understanding how the genetic architecture works with the tumor microenvironment is critical to uncovering new vulnerabilities. In addition, a thorough understanding of the interactions between different cell populations within the BM microenvironment in response to transient stress due to therapy which leads to changes in nutrient availability and metabolic adaptations, will increase the chances of a true cure.

LSCs have metabolically distinct subpopulations and relapsed/refractory LSCs switch to alternative metabolic pathways to fuel OXPHOS, rendering them therapeutically resistant to conventional treatments. Developing treatment strategies that target these metabolic pathways is key to eliminating relapsed/refractory LSC populations and improving treatment outcomes.

## DECLARATIONS

### Copyright

© The Author(s) 2023.
